# Current and Future Therapeutic Strategies for Limb Girdle Muscular Dystrophy Type R1: Clinical and Experimental Approaches

**DOI:** 10.3390/pathophysiology28020016

**Published:** 2021-05-18

**Authors:** İzem Olcay Şahin, Yusuf Özkul, Munis Dündar

**Affiliations:** Department of Medical Genetics, Medical School, Erciyes University, 38039 Kayseri, Turkey; izemolcay@gmail.com (İ.O.Ş.); ozkul@erciyes.edu.tr (Y.Ö.)

**Keywords:** LGMDR1, CAPN3, calpainopathy, therapy strategies

## Abstract

Limb girdle muscular dystrophy type R1 disease is a progressive disease that is caused by mutations in the *CAPN3* gene and involves the extremity muscles of the hip and shoulder girdle. The CAPN3 protein has proteolytic and non-proteolytic properties. The functions of the CAPN3 protein that have been determined so far can be listed as remodeling and combining contractile proteins in the sarcomere with the substrates with which it interacts, controlling the Ca^2+^ flow in and out through the sarcoplasmic reticulum, and regulation of membrane repair and muscle regeneration. Even though there are several gene therapies, cellular therapies, and drug therapies, such as glucocorticoid treatment, AAV- mediated therapy, CRISPR-Cas9, induced pluripotent stem cells, MYO-029, and AMBMP, which are either in preclinical or clinical phases, or have been completed, there is no final cure. Inhibitors and small molecules (tauroursodeoxycholic acid, salubrinal, rapamycin, CDN1163, dwarf open reading frame) targeting ER stress factors that are thought to be effective in muscle loss can be considered potential therapy strategies. At present, little can be done to treat the progressive muscle wasting, loss of function, and premature mortality of patients with LGMDR1, and there is a pressing need for more research to develop potential therapies.

## 1. Introduction

Calpainopathy is a progressive disease that causes weakness over time in the muscles that affect the upper extremities, including the hip and shoulder girdle muscles [1]. This disease, also known as limb girdle muscular dystrophy type R1 (LGMDR1; 253600 [2] or LGMD2A, as it used to be known), which is caused by defects in the calpain-3 (*CAPN3*; 114240) [2] gene, which is localized on 15q15.1, is an inherited autosomal recessive condition [3]. However, it was recently shown that there are autosomal dominant inherited variants [4]. Although the onset of the disease varies, it usually occurs in adolescence (8–15 years of age) [5]. There is a loss of ambulation in patients 10–20 years after the onset of the disease. Loss of ambulation is seen after the age of 60 in mild forms [4]. Patients exhibit muscle pain, cramps, fatigue, and exercise intolerance. Although muscle loss is symmetrical, it causes a waddling gait, wing scapula, and hyperlordosis in patients [6]. In addition, various muscle contractures are seen, especially involving the Achilles tendon [6]. According to reports in the Leiden Open Variation database as of October 2020, there are more than 490 pathogenic variants of *CAPN3* [7].

Although the function of the CAPN3 protein in cells has not been fully elucidated, studies so far have revealed the proteolytic and non-proteolytic activities of the CAPN3 protein. The full-length form of CAPN3 is expressed mainly in skeletal muscle [8]. CAPN3 demonstrates its non-proteolytic activity by stabilizing Ca^2+^-handling proteins (CSQ [9], SERCA [10], RyR1 [11], CaMKII [11], and NCX3 [12]) and maintaining Ca^2+^ homeostasis, which is extremely important and essential for muscle structure and function [5]. CAPN3 shows its proteolytic activity by targeting proteins (myostatin, titin, α-actinin-3, tropomyosin, and LIM-domain binding protein 3 [13]) that play major roles in the regulation of sarcomere stability/integrity and muscle contraction as substrates [14]. CAPN3 is responsible for muscle restructuring and function, not muscle formation [15]. Interestingly, CAPN3 also has an autodegradation property that prevents it from being detected by biochemical tests [16]. The CAPN3 protein is important for the functionalization of many proteins that it targets through its proteolytic feature. In addition, its relationship with calcium and sodium makes it indispensable for muscle cells [14]. For these reasons, CAPN3 is of critical importance in the muscle cell, not only due to its primary function but also due to its secondary functions regarding the proteins and molecules it interacts with.

## 2. Current Clinical and Experimental Studies

Among the hereditary diseases that are already difficult to treat, gene therapy for hereditary muscle diseases is an especially challenging area because of the fact that muscles make up about 40 percent of the human body. To date, mostly palliative or symptomatic treatment strategies have been presented to LGMDR1 patients. Furthermore, few experimental studies and fewer clinical study strategies have been carried out by researchers for the development of therapies in LGMDR1. In particular, the prognosis of LMGDR1 varies widely according to the location of the mutation in the *CAPN3* gene, the type of mutation, and homozygous/heterozygous status [17], such that even siblings with identical mutations might have different phenotypes and prognosis [18,19].

**Figure 1:**
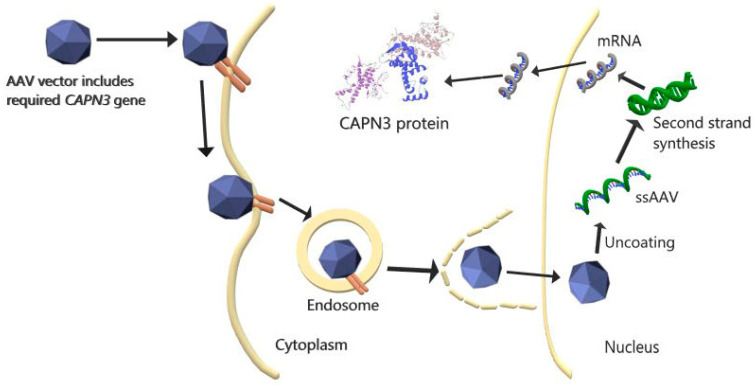
AAV-mediated *CAPN3* gene therapy. rAAV, containing the desired *CAPN3* gene, binds to the appropriate receptor and enters the cell via the endosome. AAV enters the nucleus after escaping from the endosome in the cytoplasm. After the AAV enters the nucleus and separates it from its capsid, the single-stranded (ss) DNA is transformed into double-stranded DNA, and the desired CAPN3 mRNA is transcribed by the cell. The mRNA, when leaving the nucleus and entering the cytoplasm, is translated into the CAPN3 protein [38].

An ongoing phase II trial study involving drug therapy is focused on the effect of weekly oral glucocorticoid steroid (prednisone) administration in LGMDR1 patients [20]. Various steroid applications have been successful in DMD in the past [21,22,23]. Due to the immunosuppressive properties of glucocorticoids, it is estimated that steroids may reduce muscle damage that may occur due to the inflammatory response in muscle diseases [24,25]. In another drug treatment phase I/II study, MYO-029, an antibody that neutralizes the myostatin protein, which has an inhibitory role in muscle growth, was tested in patients with various types of muscular dystrophy [26]. Although the MYO-029 drug was found to be safe, it has been determined that it is incapable of increasing muscle strength [26]. A gene therapy experimental study has also been performed concerning this therapeutic agent [27]. In that study, by performing the inhibition of myostatin via the AAV-mediated expression of a mutated propeptide (pAAV-CMV-mSeAPpropmyoD76A), researchers identified an increase in absolute power, in addition to an increase in muscle mass in CAPN3-deficient mice [27] (Figure 1). However, in a recent study, researchers who performed the genetic inhibition of myostatin by increasing the expression of follistatin, an endogenous inhibitor of myostatin in the C3KO model, reported that this intervention was not effective in developing muscle strength in proximal limb muscles, finding that it even worsened exercise intolerance and decreased the oxidative capacity of the muscle, while only increasing muscle mass 1.5–2-fold [28]. Although myostatin inhibitors are good therapeutic agents for muscle diseases, the lack of consensus in studies using myostatin inhibitors as therapeutic agents for LGMDR1 shows that this strategy has poor validity since the pathophysiology of LGMDR1 has not been completely elucidated and the exact way in which LGMDR1 makes changes is not known [29,30]. One of the gene therapy strategies designed to reverse the CAPN3 defect is to systemically or locally (intramuscularly) administer AAV-associated CAPN3 gene transfer in the murine model [31,32,33]. However, it has been reported that the increase in CAPN3 expression in extra-muscular cells due to intravenous administration causes a cardiotoxic effect, which leads to cell death and especially to heart hypertrophy [31]. In the same study, a strategy was successfully developed to overcome this toxicity by adding cardiac-specific microRNA-208a to the CAPN3 regulatory cassette in the heart to prevent CAPN3 expression [31]. In another study using a strategy with the aim of overcoming this cardiac toxicity, heart damage previously seen in the murine model was not seen in a primate model, and this strategy led to a therapeutic effect in CAPN3 deficiency [34]. In addition, mice were thought to be more susceptible to cardiac toxicity due to the difference in titin (containing binding sites of CAPN3) transcripts in the murine model compared to primates and humans [34]. Researchers have used a serotype (AAVrh74) that can target skeletal muscle and cardiac muscle without off-target delivery in the in vivo transfer of AAV in neuromuscular diseases [35,36]. A recent study tested the biodelivery and stability of this vector system in LGMDR1 by loading the CAPN3 gene into the AAVrh74 serotype with the tMCK promoter, which was applied intravenously. According to their results, the authors been stated that this vector (AAVrh74.tMCK.hCAPN3) has no off-target effects and no toxic effects and has a successful therapeutic effect even at low doses [37].

In LGMDR1 patients, the expression of FRZB, which prevents the translocation of β-catenin into the nucleus, is upregulated via inhibition of the Wnt pathway [39]. Normal CAPN3 regulates the localization of β-catenin [40]. The WNT signal is activated by its ability to activate the multimerized TCF/LEF luciferase reporter structure of 2-Amino-4-(3,4-(methylenedioxy)benzylamino)-6-(3-methoxyphenyl) pyrimidine (AMBMP), a WNT agonist that activates the canonical signal [41,42]. AMBMP activates CaMKII in metabolically altered C3K0 muscles and reprograms the muscle towards the slow oxidative muscle phenotype [43]. AMBMP reversed the LGMDR1 phenotype in vivo by improving oxidative properties, increasing slow fiber size, and improving exercise performance [43].

**Figure 2:**
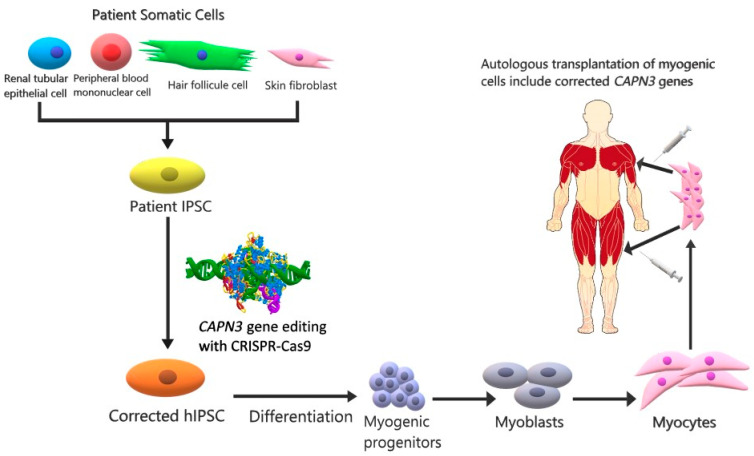
CAPN3 cell therapy with a combination of IPSCs and CRISPR-Cas9. In IPSCs derived from the somatic cells of the LGMDR1 patient (renal tubular epithelial cell, peripheral blood mononuclear cell, hair follicle cell, skin fibroblast) using reprogramming factors, the CAPN3 gene is corrected by means of the CRISPR-Cas9 method. Genetically modified IPS cells are then stimulated with various factors (Pax3/Pax7 or MyoD) to differentiate into myogenic progenitors that can be multiplied in number. Cellular therapy is applied to the patient by injecting the proliferated myocytes intramuscularly into the muscles [46].

The CRISPR-Cas9 system, which won the 2020 Nobel Prize in Chemistry, is a groundbreaking area of research for in vivo gene therapy. Using the stem cell method, a wide variety of diseases can be treated [44]. In a study combining these two strategies, muscle engenderment and increases in CAPN3 mRNA were observed in mice as a result of the transplantation of corrected LGMDR1 myogenic progenitors through the use of IPSCs, which were gene-corrected with the CRISPR-Cas9 method (Table 1) [45] (Figure 2).

## 3. Future Therapy Strategies

It is known that mitochondrial damage is involved in the pathophysiology of LGMDR1 [47,48]. A new muscle-specific protein (Mss51) has recently been identified [49]. Considering that when the gene encoding Mss51 is deleted in mice, energy production increases and mitochondrial activity improves, researchers are investigating whether the elimination of this protein would be a suitable target in a calpainopathy model [50]. Although the pathophysiology of calpainopathy has not been fully elucidated yet, some experimental and bioinformatic results show that CAPN3 targets the E3 ubiquitin proteins MuRF1 [51] and TRIM32 [52] as substrates. In addition, a recent study reported that LGMDR1 is associated with endoplasmic reticulum (ER) stress [5]. In the ER (called the sarcoplasmic reticulum in muscle cells), protein synthesis, folding, maturation and transport, calcium storage, and lipid biosynthesis take place. ER stress is defined as the disruption of the balance between the protein folding capacity of the ER and the processed protein load, resulting in the accumulation of incorrectly folded or unfolded proteins [53]. Excessive synthesis of secretory proteins, mutations in proteins that play a role in protein folding, abnormal changes in the amount of Ca^+2^ in the ER, and viral infections are some of the factors that cause protein accumulation in the ER [54,55]. It would not be wrong to say that this plays a role in the pathophysiology of LGMR1, as the deterioration of homeostasis in the ER contributes to ER stress. Considering that the perturbation of calcium flux causes ER stress, a study reported that the SERCA2 protein, responsible for the reuptake of calcium into the ER, is decreased in LGMDR1 patients [56]. In this context, it should not be ignored that targeting ER stress may be therapeutic. In order to eliminate the ER stress caused by an imbalance between the load of unfolded proteins in the ER and the capacity of the cellular mechanism that manages this load, cells activate three mechanisms. The first of these is to reduce the protein load entering the ER via a temporary adaptation by reducing the synthesis of the protein in the cell and its translocation to the ER. Secondly; the unfolded protein response (UPR) is switched on. To this end, an increase in the capacity of the ER emerges to overcome unfolded proteins for a longer-term adaptation, which requires transcriptional activation of UPR target genes. Lastly, if homeostasis cannot be restored, a cell death response occurs to protect the organism from cells displaying unfolded proteins [57]. In eukaryotes, the ubiquitin-proteosome system is responsible for most of the protein degradation in cells, aimed at maintaining protein homeostasis. UPR, pancreatic ER kinase-like (PERK), inositol requiring enzyme 1 (IRE1), and activating transcription factor 6 (ATF6) activate three important signaling pathways, initiated by stress sensors localized in the ER [58,59]. PERK phosphorylates and inactivates the eukaryotic initiation factor 2α (elF2α) with the formation of the PERK oligomer in the ER membrane [60]. Thus, mRNA transcription in the ER stops and the protein load is reduced [60]. Furthermore, when the cell encounters ER stress, ATF6 undergoes posttranscriptional modification. ATF6 sent to the Golgi apparatus undergoes clipping by interacting with site1 protease. Thus, an attempt to protect it against stress is made by increasing the ER folding capacity [61]. IRE1, which is bound to GRP78 under normal physiological conditions, becomes active through either transphosphorylation and RNAase activation or by directly binding to unfolded proteins. It stimulates the insertion of a 26-nucleotide segment from the mRNA of X-box binding protein 1 (XBP1) by cutting the activated IRE1 intron. XBP1, transformed from the spliced mRNA, eventually passes into the nucleus. XBP1 increases protein folding, ER biogenesis, and transcription of genes involved in ER-associated protein degradation (ERAD) to correct ER homeostasis [62]. As a result, through these pathways, unfolded proteins or misfolded proteins are degraded by the proteasome system or the process of cell death begins [63].

Using ER stress inhibitory agents or eliminating the causes of ER stress may be therapeutic in LGMDR1. The chemical chaperone mimetic drug tauroursodeoxycholic acid (TUDCA) has been reported in previous studies to have a reducing effect on ER stress-related molecules, such as ATF6α, IRE1α, PERK, CHOP, and GRP78 [64,65]. Salubrinal, which can be used as another therapeutic agent, can cooperate with transformational attenuation to reduce ER protein overload by inducing degradation of non-translated ER-targeted protein mRNAs [66]. Rapamycin, another agent that targets ER stress, provides the inhibition of mTORC1, triggering the autophagic process that targets toxic products, and this process provides a reduction in ER stress and a decrease in fibrosis and inflammation, in addition to an increase in contraction and strength in dystrophic muscles [67,68,69]. These results show that rapamycin may be therapeutic in muscular dystrophies.

**Figure 3:**
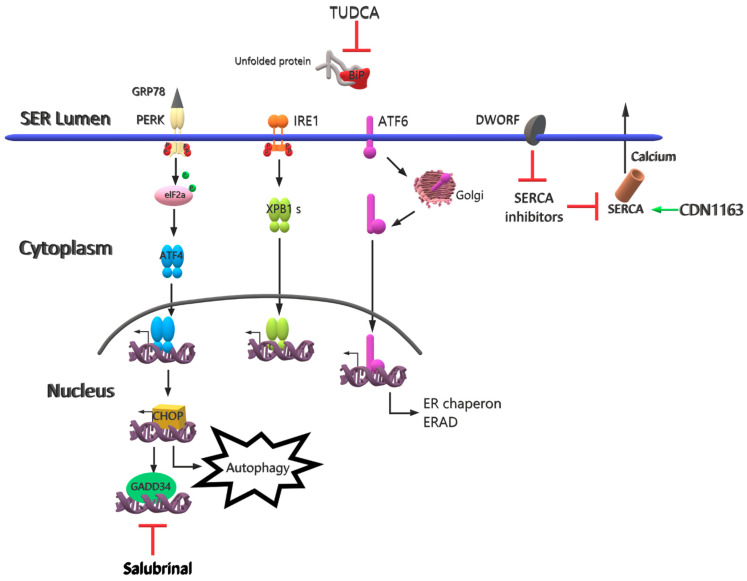
Future therapy strategies for LGMDR1. Calcium imbalance, thought to play a role in the pathophysiology of LGDMR1, can be repaired by inhibiting SERCA inhibitors through DWORF and by activating SERCA2 through CDN1163. ER stress can be reduced by targeting the degradation of proteins at the mRNA level through salubrinal and by preventing unfolded proteins associated with ER stress by means of TUDCA.

Some small molecules targeted in various diseases are preferred due to their therapeutic potential [70]. In LGMDR1, the small molecule SERCA2 activator CDN1163, which acts on the SERCA enzyme directly through the allosteric mechanism, can be used to increase the activity of SERCA2 to both reduce ER stress and maintain Ca^+2^ homeostasis [71,72]. Another molecule in which a SERCA regulatory property was discovered is a putative muscle-specific long non-coding RNA which is called dwarf open reading frame (DWORF) that encodes a 34-amino-acid peptide [73]. DWORF is localized in the SR membrane and increases SERCA activity by modifying SERCA inhibitors phospholamban, sarcolipin, and myoregine [73]. DWORF is an endogenous peptide that is known to be effective in increasing muscle contraction, and it activates the SERCA pump through a physical interaction routine (Table 2) [73] (Figure 3).

## 4. Conclusions

In conclusion, therapy strategies for LGMDR1 disease, which is caused by CAPN3 defects, continue to be developed at both clinical and pre-clinical stages. In this article, we have summarized potential therapy strategies, in addition to actual therapies, such as cell therapy, gene therapy, and drug strategies. Although gene correction is the first strategy that comes to mind in single-gene diseases, the literature offers researchers several therapeutic agents that target many factors that are effective in the pathophysiology of LGMDR1. These agents consist of inhibitors and small molecules that target ER stress, which is thought to play a role in muscle loss in LGMDR1. Although several strategies have been attempted, no definitive conclusion has yet been reached. In viral systems used in gene therapies, failure or unexpected systemic effects may occur due to delivery and serotype compatibility problems. In addition, the problem of off-target effects must be overcome. Similarly, in cell therapies, the material produced for therapy should be able to target the desired organ and perform permanent treatment there. Furthermore, the success of a drug in a similar disease does not guarantee that it will always be effective for the target disease. Disease pathophysiology is important in this context. Such problems have also been observed in the drug strategies used against LGMDR1, a disease of which the pathophysiology has not been fully clarified yet. As mentioned above, although some strategies may seem effective in achieving goals in single-gene diseases, things do not always go as expected in science. However, this should not be daunting for scientists. It should not be forgotten that the tools used in science are increasing day by day and strategies for treatment can be obtained through various combinations of tools. More researchers need to work on LGMDR1 in order to achieve the desired end and restore the health of people suffering from this disease.

## Figures and Tables

**Table 1:** Current therapy strategies.

	Type	Administration	Expectation	Stage	Comment	Ref.
Drug Therapy						
Prednisone	Glucocorticoid steroid	Taking orally	Reduce inflammatory response	Phase I/II study	Undesirable situations may occur due to suppressing the immune system.	[20]
MYO-029	Antibody	Injected intravenously	Neutralize myostatin protein	Phase I/II study	Myostatin inhibition resulted in a minor improvement in muscle.	[26]
Anti-myostatin antibody	Antibody	Injected intraperitoneally	Inhibition of follistatin, which is an endogenous inhibitor of myostatin	Experimental study on a murine model	Increase in muscle mass but not in functional muscle.	[28]
AMBMP	Small molecule	Injected intraperitoneally	As a Wnt agonist activates CaMKII	Experimental study on a murine model	Induction of slow oxidative genes.	[43]
Gene Therapy						
pAAV-CMV-mSeAPpropmyoD76A vector	Plasmid DNA	Injected intramuscularly	Inhibition of myostatin	Experimental study on a murine model	Increase in muscle mass and absolute power	[27]
CAPN3 gene transfer via AAV vector,	Plasmid DNA	Systemic injection	Replacement of functional CAPN3 gene	Experimental study on a murine model	CAPN3 overexpression caused cardiac toxicity.	[31]
CAPN3 gene, and cardiac-specific microRNA-208a transfer via AAV	Plasmid DNA	Systemic injection	Replacement of functional CAPN3 gene and overcoming cardiac toxicity	Experimental study on a murine model	CAPN3 expression and no cardiac toxicity were achieved.	[31]
AAVrh74.tMCK.hCAPN3 vector	Plasmid DNA	Injected intravenously	Replacement of functional CAPN3 gene, overcoming off-target and toxic effects	Experimental study on a primate model	CAPN3 expression, no toxicity, and skeletal-muscle-specific vector were achieved.	[37]
rAAV-C3+miRT and rAAV-C3	Plasmid DNA	Injected intravascularly and intramuscularly	Replacement of functional CAPN3 gene and overcoming cardiac toxicity	Experimental study on a primate model	In murine models, overexpression of CAPN3 is more prone to cardiac toxicity than in primates, due to physiological differences. CAPN3 expression increased in both applications and no cardiac toxicity was observed.	[34]
Combined Therapy (Cell- and Gene-Based)						
IPSCs	CRISPR-Cas9 and stem cell	Injected intramuscularly	Replacement of functional CAPN3 in myogenic progenitor and mature muscle cells expressing CAPN3	Experimental study on a murine model	CAPN3 mRNA levels were increased.	[44]

**Table 2:** Future therapy strategies.

	Type	Application	Expectation	Ref.
Mss51	Muscle-specific protein	Inhibition of Mss51 gene	Energy production increases and mitochondrial activity improves	[50]
TUDCA	The chemical chaperone mimetic drug	Different applications of TUDCA	Reduces effects on ER stress-related molecules	[65]
Salubrinal	A small molecule for selective inhibition of eIF2α	Different applications of salubrinal	Induces degradation of non-translated ER-targeted protein mRNAs	[66]
Rapamycin	Drug	Oral gavage	Provides inhibition of mTORC1, decrease in ER stress and inflammation, Improves muscle strength	[67]
CDN1163	A small molecule as a SERCA2 activator	Injected intraperitoneally	Reduces ER stress and maintains Ca^+2^ homeostasis	[71]
DWORF	Muscle-specific long non-coding RNA	Upregulate of DWORF gene	Inhibits SERCA inhibitors and increases SERCA activity	[73]

## Data Availability

Not applicable.

## Abbreviations

AAV	Adeno-associated virus
AAVrh74.tMCK.hCAPN3	AAV rhesus 74 truncated muscle creatine kinase human CAPN3
AMBMP	2-amino-4-(3,4-(methylenedioxy)benzylamino)-6-(3-methoxyphenyl) pyrimidine
ATF6	Activating transcription factor 6
C3KO	Calpain 3 knock out
CaMKII	Ca^2+^/calmodulin-dependent protein kinase II
CaMKII	Ca^2+^/calmodulin (CaM)-dependent protein kinase II
CAPN3	Calpain 3
Cas9	Native Cas9 nuclease
CHOP	C/-EBP homologous protein
CRISPR-Cas9	Clustered regularly interspaced short palindromic repeats CRISPR-associated proteins 9
CSQ	Calsequestrin
DMD	Duchenne muscular dystrophy
elF2α	Eukaryotic initiation factor 2α
ER	Endoplasmic reticulum
ERAD	ER-associated protein degradation
FRZB	Frizzled-related protein
GRP78	Glucose-regulated protein
IPSC	Induced pluripotent stem cell
IRE1α	Inositol-requiring enzyme 1α
LGMDR1	Limb girdle muscular dystrophy R1
LIM	Lin-11 Isl-1 Mec-3
Mss51	Mitochondrial translational activator
MuRF1	Muscle RING-finger protein-1
MYO-029	Stamulumab
MyoD	Myogenic differentiation antigen
NCX3	Na^+^-Ca^2+^ exchanger 3
pAAV-CMV-mSeAPpropmyoD76A	Plasmid AAV-cytomegalovirus- murine-secreted alkaline phosphatase myogenic differentiation antigen murine-secreted alkaline phosphatase
Pax3/Pax7	Paired box gene 3/Paired box gene 7
PERK	PKR-like ER kinase
RyR1	Ryanodine receptor 1
SERCA	Sarcoplasmic/endoplasmic reticulum Ca^2+^ ATPase
TCF/LEF	T-cell factor/lymphoid enhancer factor
TRIM32	Tripartite motif-containing protein 32
TUDCA	Tauroursodeoxycholic acid
UPR	Unfolded protein response
WNT	Wingless-related integration site
XBP1	X-box binding protein 1
